# Analysis of inhibitor of apoptosis protein family expression during mammary gland development

**DOI:** 10.1186/1471-213X-10-71

**Published:** 2010-06-28

**Authors:** Thomas W Owens, Fiona M Foster, Jolanta Tanianis-Hughes, Julia Y Cheung, Lisa Brackenbury, Charles H Streuli

**Affiliations:** 1 Wellcome Trust Centre for Cell-Matrix Research, Faculty of Life Sciences, University of Manchester, Manchester, M13 9PT, UK

## Abstract

**Background:**

Inhibitors-of-Apoptosis-Proteins (IAPs) are an evolutionarily conserved family of proteins capable of regulating several facets of apoptosis. IAPs are frequently dysregulated in cancer, but their role in the regulation of apoptosis during developmental processes is not fully understood. Here we examined the expression of IAPs during the post-natal development of the mouse mammary gland, which is a tissue that exhibits a profound induction of apoptosis during involution.

**Results:**

Six out of eight mammalian IAP family members are expressed in the mammary gland. Notably, quantitative PCR and immunoblotting revealed that XIAP, c-IAP1 and c-IAP2 are down-regulated in pregnancy and lactation, and prior to the onset of involution. In cultured mammary epithelial cells (MECs), XIAP levels decreased in response to inhibition of growth factor signalling. Maintaining XIAP levels in MECs by expressing exogenous XIAP protected them from all apoptotic stimuli tested.

**Conclusions:**

These data suggest that the developmental regulation of IAP expression *in vivo *contributes to naturally occurring programmes of cell death.

## Background

Dysregulated apoptosis is a feature of cancer, where apoptosis resistance promotes tumour progression by giving cancer cells a survival advantage. For example, resistance to apoptosis induced by loss of adhesion signals allows cancer cells to metastasise [1,2]. Moreover, intrinsic and acquired resistance to apoptosis are barriers to successful cancer treatments. Understanding the mechanisms that control apoptosis under normal developmental settings is important in order to provide opportunities for designing novel anti-cancer therapeutics.

The mammary gland provides a paradigm to study mechanisms regulating developmental apoptosis [2-5]. During cycles of mammary gland development, the differentiated epithelial cells that produce milk in lactation undergo widespread apoptosis after weaning, as the gland involutes and remodels to a pre-pregnant state. Elucidating the mechanisms that regulate the sensitivity of mammary epithelial cells (MECs) to apoptosis will provide insight into possible breast cancer targets [6,7]. Currently the molecular basis of mammary involution is not fully understood. Here we have examined the expression and possible role in mammary gland development of a central family of apoptosis regulators, the Inhibitors-of-Apoptosis-Proteins (IAPs).

IAPs are endogenous apoptosis regulators, though recently they have been shown to have additional diverse roles in cell regulation [8-11]. IAPs are evolutionarily conserved from yeast to humans and are characterised by the presence of one or more baculovirus IAP repeat (BIR) domains. The BIR domains target IAPs to bind and inhibit caspase function [8,12]. During cell death, the natural anti-apoptosis function of IAPs is overcome via competition for their caspase-binding sites by Smac and Omi, as well as by ubiquitination [13-15].

The 8 mammalian family members exhibit distinct patterns of tissue expression, however almost nothing is known about their expression and function during normal mammary gland development, although they are acknowledged to be frequently dysregulated in breast cancer [16,17].

Using quantitative PCR and immunoblotting we examined IAP family member expression during post-pregnancy mammary gland development, and discovered that several IAPs are down-regulated prior to the gland entering involution. We suggest that cell-autonomous regulation of IAP expression might have a central role in sensitising MECs for apoptosis that occurs during involution of the tissue.

## Results

### IAP expression during mammary gland development

**Figure 1:**
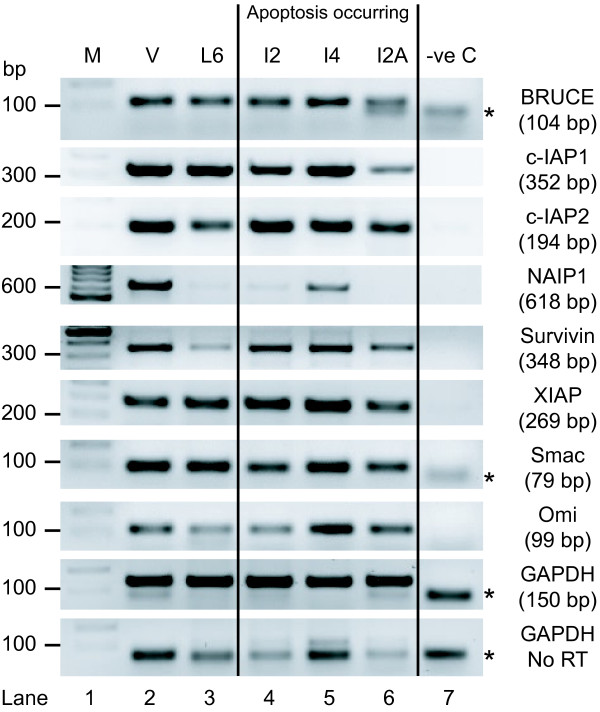
**IAP family members expressed during mammary gland development**. Total RNA extracted from mammary gland tissue of virgin (V), lactation day 6 (L6), involution day 4 (I4) and 2 × involution day 2 (I2 & I2A) mice was subjected to reverse transcription. Control reactions were performed simultaneously in which the reverse transcriptase (RT) was omitted. cDNA was analysed by PCR with primers specific to each IAP (expected PCR product size is shown in brackets). GAPDH primers were used as a positive control (M - DNA ladder, -ve C - no cDNA, *- primer dimer products).

Initial studies using RT-PCR were performed to identify which IAPs are expressed in the mammary gland. BRUCE, c-IAP1, c-IAP2, NAIP1, Survivin and XIAP were detected in mouse mammary gland at the time points examined (Figure 1). Livin cDNA was not detected at any of the time points, suggesting that it is not expressed in the mammary gland (data not shown). The IAP antagonists, Smac and Omi, were also present. Thus most of the known IAPs and their antagonists are transcribed in the mammary gland and are present throughout gland development. Since RT-PCR does not reveal changes in levels of RNA, we performed qPCR analysis. XIAP, c-IAP1 and c-IAP2 were chosen for subsequent analysis because they have roles in breast cancer progression [18].

The transition from lactation to involution marks the period in development during which substantial and synchronous induction of apoptosis occurs. We hypothesised previously that the epithelial cells in lactating mammary gland might become primed for rapid apoptosis by alterations in the levels of apoptosis regulators during lactation [19]. To determine whether the levels of IAPs changed from pregnancy to lactation and/or during involution, we carried out qPCR analysis between the end of pregnancy and 72 hours of involution. During this time the predominant cells are MECs, and these are the cells that undergo apoptosis at involution. In the mouse, cell death begins within 24 hours of involution, and tissue remodelling occurs from ~72 hours [20].

**Figure 2:**
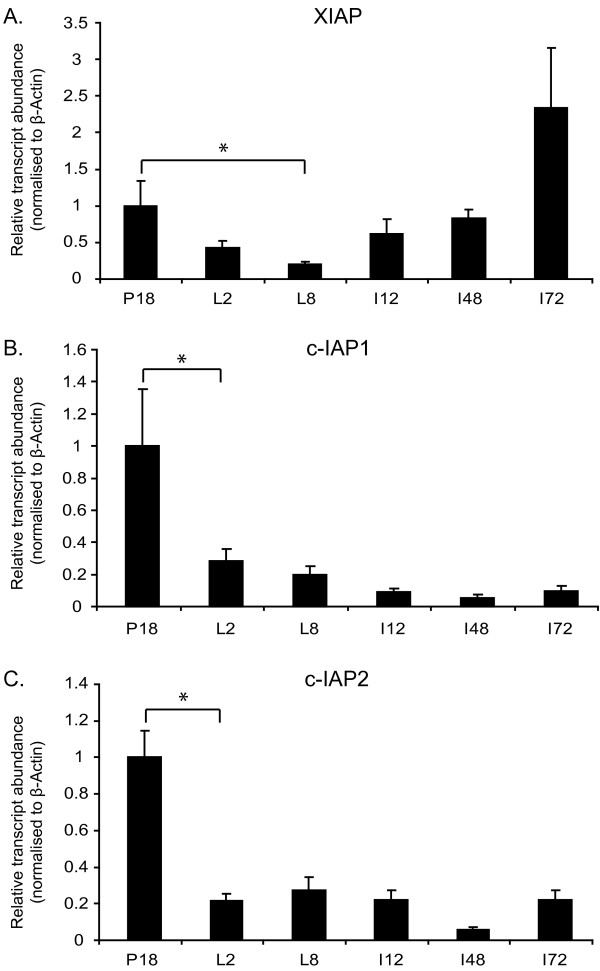
**Quantitative PCR analysis of IAP expression during mammary gland development**. cDNA produced from mammary glands isolated at the development time points shown (P - pregnancy (days); L - lactation (days) and I - involution (hours)) was analysed by quantitative PCR. β-actin was used as the internal control mRNA to which IAP transcript levels were normalised. The changes in IAP expression levels are shown relative to IAP expression in the pregnancy day 18 sample (*indicates p < 0.05) (**A**) XIAP (**B**) c-IAP1 (**C**) c-IAP2. Data shown represents the average (+/- S.E.M.) of 3 mice at each time point.

The levels of XIAP mRNA decreased between pregnancy day 18 and lactation day 8. As the gland entered involution, the relative XIAP transcript abundance remained low during the initial phase of cell death (i.e. up to 48 hours of involution) and then returned to the level observed at pregnancy day 18 as tissue remodelling began (i.e. from 72 hours of involution) (Figure 2A). c-IAP1 and c-IAP2 transcript levels were highest at the end of pregnancy (P18) and then decreased considerably as the glands entered lactation. The levels then decreased further as the gland entered involution, with expression been lowest at 48 hrs of involution (Figure 2B, C).

**Figure 3:**
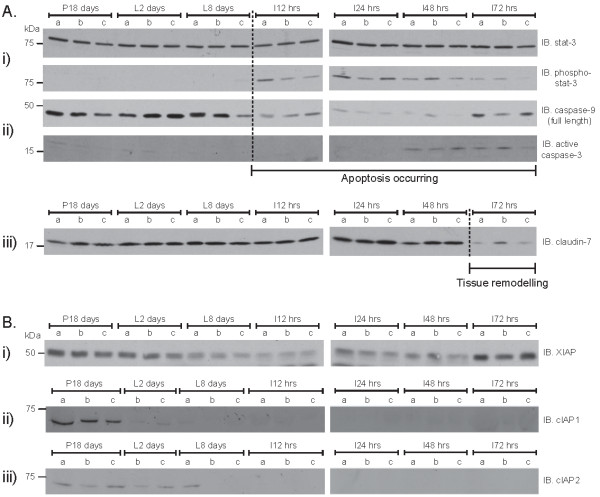
**Protein expression during mammary gland development**. Mammary gland total protein from 3 mice (a, b, and c) was immunoblotted with the antibodies shown (P - pregnancy (days); L - lactation (days) and I - involution (hours)). (**A**) Phospho-stat3 is a marker of the onset of involution. Blot was stripped and re-probed with total stat-3 as a loading control (i). Full-length caspase-9 represents the inactive zymogen. Cleaved caspase-3 represents the active form (ii). Claudin-7 is a loading control for epithelial cell content between P18 days and I48 hours (iii). (**B**) Membranes were probed for XIAP, cIAP1 and cIAP2.

To determine if the changes in IAP expression at the RNA level reflected changes at the protein level, we examined IAP expression by immunoblotting. STAT3, caspases and claudin-7 were used as markers for the key developmental stages in mammary gland development. Activation of the transcription factor STAT3 immediately follows the cessation of suckling and is required for the onset of involution [21]. STAT3 phosphorylation was not evident during pregnancy and lactation, but occurred within 12 hours of involution and remained for at least the following 72 hours (Figure 3Ai). Caspases are activated during apoptosis and are responsible for cell degradation [22]. Expressed as inactive zymogens, caspase cleavage (i.e. loss of full-length protein, or appearance of cleavage fragments) indicates that activation has occurred. Caspase-9 levels decreased at involution 12 hours and remained low until involution 48 hours; caspase-3 is activated by caspase-9 and its cleaved form was detected at involution 48 and 72 hours (Figure 3Aii). Finally, claudin-7 is an epithelial tight junction component expressed by mammary epithelial cells and thought to be involved in regulating cell-ECM interactions [23]. Its levels remained equal from pregnancy through to 48 hours of involution, and then showed a marked decrease as the gland entered the tissue remodelling stage (Figure 3Aiii).

Thus our samples represent stages in the progression of mammary gland involution; STAT3 and caspase-9 become activated within 12 hours; death becomes maximal at around 48-72 hours with high levels of caspase-3 activated; and the tissue remodeling begins at 72 hours post-weaning. We next examined IAP expression during this time course.

XIAP protein levels remained constant between the end of pregnancy and early lactation, but decreased at lactation day 8 (Figure 3Bi). XIAP protein levels remained low until involution 72 hours when they then returned to a pre-lactational level. Thus, XIAP protein is expressed at low levels in the late lactating mammary gland prior to the onset of cell death. The protein profile is similar to the mRNA level, but does not follow it precisely, suggesting that XIAP is regulated by both RNA and protein processing during post-pregnancy mammary gland development.

c-IAP1 protein expression was highest at pregnancy day 18 and decreased considerably by lactation day 2 (Figure 3Bii). In contrast, c-IAP2 levels remained unchanged between pregnancy day 18 and lactation day 2, but became undetectable by the time that the gland entered involution (Figure 3Biii). Expression of both c-IAP1 and c-IAP2 then remained undetectable throughout involution. The protein level of c-IAP1 closely matched the qPCR profile, suggesting that c-IAP1 expression is regulated at the level of transcription in post-pregnancy development. Although the changes in transcript abundance of c-IAP2 closely matched that of c-IAP1, the c-IAP2 protein level decreased later on in lactation, which implies that the c-IAP1 and c-IAP2 proteins are subjected to different modes of regulation in the mammary gland.

**Figure 4:**
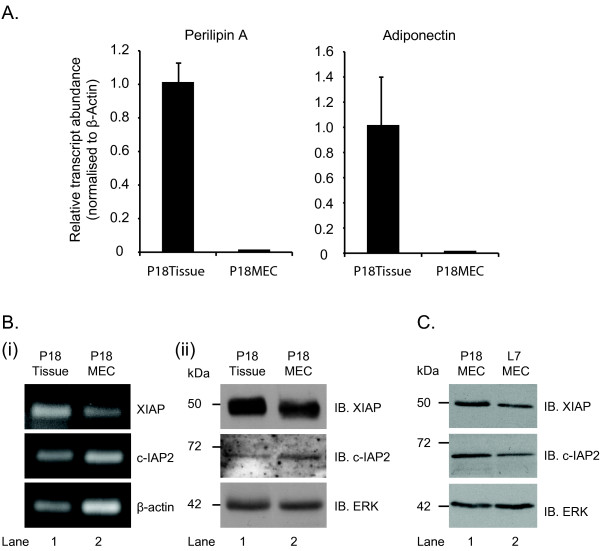
**IAP expression in purified MECs**. Mammary epithelial cells were purified from the combined mammary gland tissue of 2 mice at the time points shown (P18 - pregnancy day 18, L7 - lactation day 7). (**A**) Quantitative PCR analysis of the levels of adipocyte-specific gene expression in whole tissue lysate (Tissue) and purified mammary epithelial cells (MEC). (**B**) Analysis of IAP expression in P18Tissue and P18MEC. (i) RT-PCR of mRNA extracted from P18Tissue and P18MEC was performed using primer pairs targeted against each IAP. (ii) Protein lysates were immunoblotted with antibodies shown. (**C**) IAP expression in purified MECs extracted from mice at P18 and L7 was analysed by immunoblotting.

Finally, to determine whether the changes in IAP expression observed *in vivo *were due to altered expression in the mammary epithelial cells, rather than reduced levels of stromal components such as adipocytes, we examined IAP levels in primary MECs immediately after isolation from the intact tissue. Quantitative PCR analysis using primers targeted against the adipocyte-specific genes Perilipin A and Adiponectin, demonstrated that the purification of P18MECs had successfully removed any adipocytes that are present in the mammary gland tissue at this time (P18Tissue) (Figure 4A). RT-PCR and immunoblotting analysis showed that XIAP and cIAP2 were indeed expressed in P18MECs (Figure 4B). We next compared IAP expression in MECs isolated from pregnancy day 18 and lactation day 7 mice (P18MEC and L7MEC respectively). Consistent with our *in vivo *data, both XIAP and c-IAP2 levels were lower at lactation day 7 compared with pregnancy day 18 (Figure 4C). Together, these data confirm that expression of IAPs in mammary epithelial cells themselves decreases as the gland approaches involution.

Importantly, our results show that down-regulation of IAPs *in vivo *precedes initiator caspase activation. Because IAPs promote cell survival, the data support a model in which the mammary gland becomes primed for involution during the prior developmental stage of lactation.

### XIAP down-regulation occurs in primary MEC cultures

Since XIAP is a potent inhibitor of caspases, we hypothesised that its down-regulation might be a mechanism to prime cells *in vivo *for subsequent apoptosis. It is unlikely that XIAP down-regulation alone would cause apoptosis as XIAP null mice are viable. To test this hypothesis, we determined if XIAP down-regulation is caused by factors that promote apoptosis in MECs.

**Figure 5:**
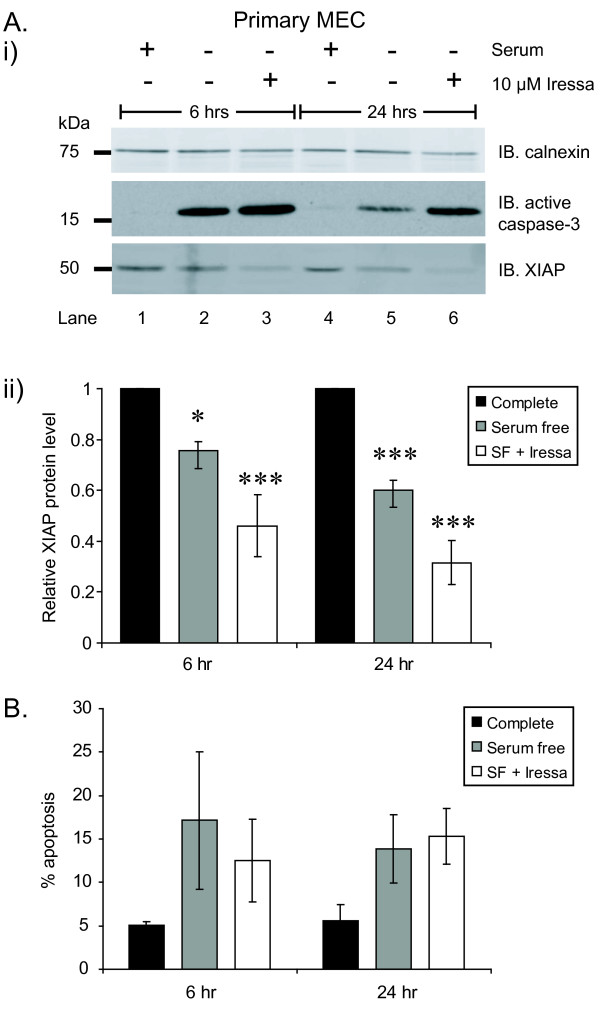
**XIAP down-regulation in response to growth factor withdrawal**. Primary mammary epithelial cells (MECs) were cultured in completed media (lanes 1 and 4), serum starved (lanes 2 and 5) or serum starved in the presence of 10 μM Iressa (lanes 3 and 6). (**A**) Whole cell lysates were immunoblotted with antibodies shown (i). XIAP protein levels relative to calnexin were calculated using Li-cor Odyssey system (ii). (**B**) Apoptosis was scored by counting the percentage of apoptotic nuclei. Results shown represent the average of 3 experiments +/- S.E.M. (*indicates p < 0.05; *** indicates p < 0.01).

One factor contributing to apoptosis induction in the mammary gland is suppression of growth factor signalling [5]. We therefore cultured primary MECs in the absence of growth factors, and in the presence of the EGFR tyrosine kinase inhibitor, Iressa [24,25]. Cleaved caspase-3 was detected within 6 hours of growth factor withdrawal (Figure 5Ai). At this time, ~20% of the cells were apoptotic (Figure 5B). Addition of Iressa to the growth factor-free media had no further effect on apoptosis, indicating that growth factor withdrawal is sufficient to induce apoptosis.

The loss of growth factor signalling caused XIAP levels to decrease by ~25% and ~40% after 6 and 24 hours, respectively (Figure 5Aii). Although Iressa did not elevate apoptosis, it caused a greater decrease in the XIAP levels compared with growth factor withdrawal alone; 55% and 70% after 6 and 24 hours, respectively. These data demonstrate that XIAP is down-regulated during the course of growth-factor withdrawal-induced apoptosis in MECs.

Since XIAP down-regulation coincided with caspase activation, it was important to examine whether it was dependent or independent of active caspases. Treatment of serum-deprived MECs with the pan-caspase inhibitor zVAD did not rescue the decline in XIAP levels seen after serum withdrawal (Additional file 1A, compare lanes 2 & 3, and Additional file 1B). Moreover, neither EGF nor insulin prevented XIAP down-regulation, even though they protected MECs from apoptosis, as judged by the levels of active caspase 3 (Additional file 1A, compare lanes 2, 4 and 5).

Together, these data indicate that the decline in XIAP levels is independent of caspase activity, and cannot be rescued by EGF or insulin/IGF signalling.

Interestingly, we found that XIAP down-regulation was a common feature of MEC apoptosis, as withdrawal of ECM-dependent survival signals (which also occurs during involution) and the kinase inhibitor staurosporine (STS), both induced XIAP down-regulation (Additional file 2).

### XIAP protects mammary epithelial cells from apoptosis

Since the XIAP protein level decreased prior to mammary gland involution, as well as in primary MEC cultures following serum withdrawal, we reasoned that XIAP reduction might contribute to the apoptosis execution programme, rather than being a consequence of it.

To test this hypothesis, we used the MEC line FSK7 (which can be transfected more efficiently than primary MEC), to determine if exogenous XIAP could protect cells from apoptosis induced by the various stimuli. FSK7 cells behaved similarly to primary MEC regarding apoptosis and XIAP down-regulation in response to all apoptotic stimuli tested (Additional file 3) [25,26].

**Figure 6:**
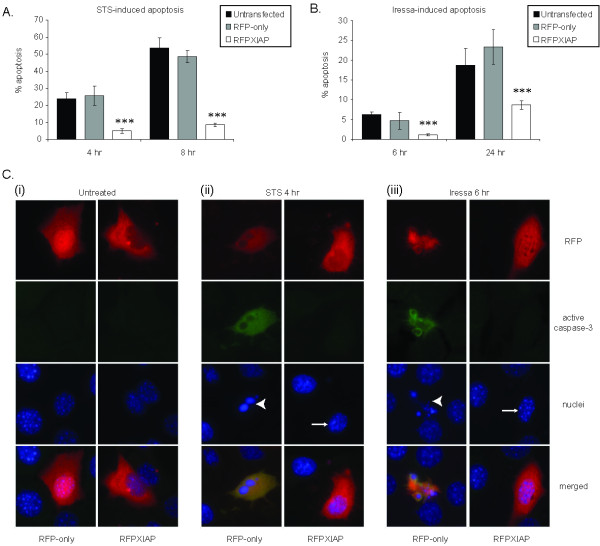
**XIAP protects MECs from apoptosis**. FSK7 cells transiently expressing monomeric RFP or RFPXIAP were treated with staurosporine (STS) for 4 or 8 hours cells, then fixed and stained with DAPI. (**A**) Apoptosis was scored as the percentage of cells with apoptotic nuclei. (**B**) FSK7 cells transiently expressing monomeric RFP or RFPXIAP were serum starved in the presence of 10 μM Iressa for times shown and percentage apoptosis scored. Data shown represents the average of 3 independent experiments +/- S.E.M. (*** indicates p < 0.01). (**C**) Representative images of mRFP and RFPXIAP expressing cells (left panels and right panels, respectively) that were either left untreated (i), treated with STS for 4 hours (ii) or treated with Iressa for 6 hours (iii). Arrows and arrowheads indicate non-apoptotic and apoptotic nuclei, respectively. Note that the RFPXIAP-expressing drug treated cells are negative for active caspase 3 and have non-apoptotic nuclei. In contrast RFP-expressing cells are stained positive for active-caspase 3 and have condensed nuclei.

Cells were transfected with either RFP alone or RFP-tagged XIAP (RFPXIAP) and then treated with apoptosis inducer. Cells treated with STS showed the expected increase in apoptosis after 4 and 8 hours, which was rescued by RFPXIAP (Figure 6A, C). Similarly, RFPXIAP prevented apoptosis induced by Iressa (Figure 6B, C) [24-26].

These data demonstrate that XIAP is capable of inhibiting apoptosis induced by a variety of stimuli. Furthermore, they suggest that the down-regulation of XIAP contributes to rapid execution of the apoptotic programme. This conclusion is supported by other studies where we have shown that reducing XIAP levels by siRNA does not directly induce apoptosis in MECs, rather it sensitises cells to apoptosis inducers [18].

## Discussion

In this study, we have demonstrated that the IAP family members; BRUCE, c-IAP1, c-IAP2, XIAP, NAIP-1 and Survivin are all expressed in the mouse mammary gland, and that XIAP, c-IAP1 and c-IAP2 are differentially expressed at different stages during the post-pregnancy development of the tissue. Moreover in cultured MECs, XIAP levels decrease in response to apoptosis stimuli, while exogenous XIAP protects MECs from apoptosis.

Of particular interest is that expression of XIAP, c-IAP1 and c-IAP2 all decrease during lactation, prior to the onset of apoptosis at involution. These data support our previous work on the levels of Bcl-2 family proteins, where we showed that the potent anti-apoptotic proteins, Bcl-2, Bcl-XL and Bcl-w all decreased during lactation and conversely, that the levels of pro-apoptotic proteins Bak and Bad increased [19]. It is important to stress that these global changes in expression of apoptosis regulators in the mammary gland occur prior to the induction of apoptosis at involution. We therefore propose that major changes occur in the apoptosis machinery during lactation in order to prepare epithelial cells for rapid execution as the mammary gland enters involution. These changes in the expression profiles of apoptosis regulators in the absence of cell death indicates that cells employ multiple levels of control beyond *bona fide *cell death regulators to govern their fate [27]. Currently, we do not know what the mechanisms are that lead to altered IAP levels, though they occur through changes in both mRNA and protein levels. A current focus is to explore the relative roles of transcriptional control as well as protein stability in regulating IAPs in the mammary gland.

Our data also provide insight into the lack of any overt apoptosis phenotype in XIAP, c-IAP1 and c-IAP2 null mice, as it shows that cells can withstand the loss of one or more of these apoptosis regulators and remain viable. However, IAP regulation does control the sensitivity of cells to death stimuli, and we suggest that decreasing IAP levels during lactation may sensitise the cells to the pro-apoptotic signals they will receive as involution is initiated [28]. This idea is supported by other cases where changes in IAP expression correlate with changes in sensitivity to apoptosis [29,30].

The onset of mammary involution is determined by the co-ordinated regulation of multiple signalling pathways [5,31]. In addition to down-regulating pro-survival proteins (as we have shown), many pro-apoptotic proteins, such as death receptors and associated ligands (i.e. Fas, FasL and TNFα) are upregulated at the onset of involution [32,33]. We suggest that altered levels of IAPs might influence the efficiency of their apoptosis signalling.

Recently, XIAP has been shown to regulate cell sensitivity to Fas-induced apoptosis [34,35]. Fas activates the extrinsic (death receptor) apoptosis pathway leading to caspase-8 activation. In most cells (known as type II) this is insufficient to kill the cell unless the intrinsic (mitochondrial) pathway is activated by caspase-dependent cleavage of Bid to form tBid. However, XIAP null cells can be killed by Fas even in the absence of Bid. Fas and its ligand FasL are both expressed in mammary gland by involution 12 hours, thus reduced XIAP expression might allow a more robust response to the Fas death signal [32,33]. Indeed, we have previously demonstrated that lowering XIAP levels in MECs by siRNA dramatically enhances their apoptotic response to the related ligand TRAIL [18].

TNFα expression is also induced early during involution [3]. Interestingly, TNFα can activate either pro-survival or pro-apoptotic signals, but it is not known how mammary epithelial cells co-ordinate the response to TNFα. c-IAP1 and c-IAP2 regulate cell sensitivity to TNFα-induced apoptosis, with the down-regulation of c-IAPs associated with a pro-apoptotic response [36,37]. Thus MECs may down-regulate c-IAPs during lactation to ensure a pro-apoptotic response to TNFα when the signal is received in involution.

Approximately 90% of mammary epithelium dies during involution. Most of these cells are alveolar MECs; however the ductal MECs survive to re-populate the gland in subsequent pregnancies. It is not known what determines which cells survive involution, but the intense pro-apoptotic signalling at this time suggests that they have increased resistance to apoptotic signals. Interestingly, at 72 hours of involution XIAP mRNA and protein expression return to pre-lactational levels. The mechanism for this increase in XIAP expression is not known. One possibility is that it may be due to increased NF-κB signalling at this time during development, as XIAP is a target of NF-κB [38,39]. Of note, NF-κB can inhibit apoptosis in MECs, and therefore it is possible that NF-κB mediates its protection on the mammary epithelium in part through up-regulation of XIAP. Conversely, XIAP can activate NF-κB signalling and thus the increase in XIAP during involution could represent part of a pro-survival feedback that protects the remaining epithelium from undergoing apoptosis [40].

Many breast cancer cells display resistance to apoptotic stimuli and interestingly, also have elevated IAP expression [18]. Thus, it will be important in the future to determine if ductal MECs retain high IAP expression levels as a mechanism of evading death during involution, as this may provide insight into breast cancer formation.

## Conclusions

Cells have developed multiple mechanisms to down-regulate IAPs during apoptosis, indicating that removing IAPs is advantageous to ensure that apoptosis occurs. Our model of involution is that terminal differentiation of luminal cells may include changes in transcription and post-translational control of apoptosis-regulating proteins, to allow the cells to be efficiently cleared from the gland by apoptosis during involution. We would therefore argue that the down-regulation of IAPs and Bcl-2 proteins prior to involution in the mammary gland, is a developmental mechanism that promotes the efficient execution of unwanted cells at weaning.

## Methods

### Reagents

Unless otherwise stated chemical reagents were obtained from Sigma.

### Antibodies

Monoclonal anti-XIAP (clone 2F1) was from Bioquote. Anti-claudin 7 was from Zymed. Anti-calnexin (C4731) was from Sigma. Anti-cleaved caspase-3, anti-Stat 3, anti-caspase 9 and polyclonal anti-XIAP were from Cell Signaling Technology. Anti c-IAP2 was from Abcam. Anti-cIAP1 was a generous gift from J Silke [36]. HRP-conjugated secondary antibodies were from Jackson ImmunoResearch Laboratories. IR dye-conjugated secondary antibodies were from Molecular Probes and Rockland Laboratories.

### Tissue extracts

All experiments are carried out in accordance with the Animal scientific procedure act (ASPA 1986), governed by the home office within the United Kingdom, each project has to be passed by our local ethical review process (LERP) before final acceptance from the home office.

Virgin ICR mice were mated at 8 weeks of age. Litters were normalised to 8 pups/mother. At 10:00 am on lactation day 8, pups were removed and involution time points were defined as the number of hours after pup removal. The mice were euthanised by CO_2 _overdose. All samples were collected at between 10:00 am - 11:00 am (involution 12 h at 10:00 pm) to reduce possible variations caused by circadian rhythms. Samples from 3 mice were collected at pregnancy day 18, lactation days 2, 6 and 8, and involution 12, 24, 48 and 72 hours. Both 4th (inguinal) glands were combined and the lymph nodes were discarded. Tissues were immediately snap frozen in liquid nitrogen, processed using a biopulveriser, and the resultant powdered tissue used to extract RNA or protein.

### Preparation of primary MECs

Primary MECs were prepared according to published procedures [41]. Tissue culture dishes were prepared, prior to mammary cell preparation. Stock rat-tail type-I collagen in 0.1% acetic acid was diluted in PBS to a final concentration of 10 μg/ml, and then used to coat the dishes which were then incubated overnight at 4°C. Collagen coated dishes were washed three times in PBS and conditioned with serum/fetuin mix (1 mg/ml fetuin in Ham's F12 sterile filtered (0.45 μM filter (Nalgene)) and supplemented with 20% FBS (Cambrex), 100 μg/ml gentamycin, 200 U/ml penicillin, 200 μg/ml streptomycin, 0.5 mg/ml fungizone 20 ng/ml EGF, 10 μg/ml insulin and 2 μg/ml hydrocortisone) for a minimum of 1 hour at 37°C, prior to plating cells.

Mammary epithelial cells were isolated from pregnancy day 14.5-18.5 ICR mice. The mice were euthanised by CO2 overdose. With care taken to avoid muscle and tendons, all ten glands were removed and the tissue placed Ham's F12 medium. The mammary glands were finely chopped with scalpels on a Teflon board. Chopped tissue was digested for 1.5 hours at 37°C with agitation in 70 ml of sterile filtered collagenase mix (14 mM NaHCO3, 10 mM HEPES, 9.8 mg/ml nutrient mixture Ham's F12, 1.5 mg/ml porcine trypsin (Gibco), 3 mg/ml collagenase B (Roche) 5% FBS). The digested tissue was centrifuged (16 × g, 1 min) to remove any large lumps of undigested tissue.

Mammary epithelial cells were then isolated as whole alveoli, excluding fibroblasts and single cells. The supernatant from the 16 × g, 1 min centrifugation was re-centrifuged (110 × g, 3 min). The resulting pellet was re-suspended in ice cold Ham's F12 and then washed three times by re-suspending in Ham's F12 and centrifugation (110 × g, 3 min). The final cell pellet was re-suspended and combined in Ham's F12 to a final volume of 50 ml. The mammary epithelial cells were then plated out onto the pre-prepared collagen I coated dishes using Ham's F12 to make up to necessary final plate volume. After 2 days post-plating the medium was replaced with complete primary medium (10% FBS (Cambrex), 50 μg/ml gentamycin, 100 U/ml penicillin, 100 μg/ml streptomycin, 0.25 mg/ml fungizone 10 ng/ml EGF, 5 μg/ml insulin and 1 μg/ml hydrocortisone).

Isolation of purified MECs for IAP expression analysis was performed using a method similar to Rudolph *et al *2009 [42]. In brief, purification of P18MECs was performed as above, except that prior to plating, the cells were either lysed directly in chilled lysis buffer (50 mM Tris-HCl, pH 7.5, 150 mM NaCl, 1 mM EDTA, 1% Nonidet P-40, 0.5% sodium deoxycholate, 0.1% SDS), supplemented with protease and phosphatase inhibitors (Calbiochem) for protein analysis, or lysed in TriZol reagent (Invitrogen) for RNA extraction. The purified MECs were isolated from combined tissue extracted from two mice at each time point.

### RNA extraction and cDNA synthesis

Total RNA was extracted using TriZol reagent (Invitrogen), treated with DNAase I (Ambion), and integrity was checked by agarose gel electrophoresis. To confirm that the RNA samples were not contaminated with DNA, PCR reactions were performed with primers to non-transcribed regions of the mouse β-actin gene. RNA samples (2.4 μg) were reverse transcribed by RevertAid First Strand cDNA Synthesis Kit (Fermentas) using random hexamers.

### Quantitative PCR (qPCR)

Primers were selected to contain minimal intra-and inter-primer interactions using Vector NTI 7 software (Additional files 4 and 5). Specificity was determined by BLAST sequence alignment searches using http://www.ncbi.nih.gov/BLAST and http://www.ensembl.org. Primer pairs that produced a single product under optimised conditions were used for quantitative analysis.

Reactions were performed using the qPCR Core Kit for SybrGreen I (RT-SN10-05, Eurogentec). The concentrations of MgCl_2 _and primers were optimised for each target sequence. Relative quantification of gene expression was performed using StepOne Plus Real Time PCR System and StepOne Software v2 (Applied Biosystems).

Standard curves were prepared using 1:2 - 1:1000 dilutions of pregnancy day 18 (sample B) cDNA. Two standard curves were prepared, one for the endogenous reference gene β-actin and one for the target gene. To ensure that reactions produced a single PCR product, melt curve analyses were performed on each reaction (and the reaction products were visualised by agarose gel electrophoresis). The melt curve was generated by heating the reaction from 72°C to 90°C with fluorescence read every 1°C.

### Data analysis

Pregnancy day 18 (sample B) was selected as the standard time point to which the relative expression levels of the other time points were compared. The relative transcript abundance of the genes of interest was obtained by normalising to the transcript abundance of β-actin, which we had validated to be a suitable normalisation gene. The StepOne software then calculated the relative transcript abundance of the genes of interest in the other samples compared with P18 (sample B).

### Cell culture

Primary MECs were isolated and cultured on collagen coated dishes as described above [43]. The XIAP protection study was done using FSK7 cells [44].

### Immunoblotting

Lysis of tissues or cells was performed using RIPA buffer (50 mM Tris-HCl, pH 7.5, 150 mM NaCl, 1 mM EDTA, 1% Nonidet P-40, 0.5% sodium deoxycholate, 0.1% SDS), supplemented with protease and phosphatase inhibitors (Calbiochem). Protein samples were separated by SDS-PAGE and then transferred to nitrocellulose membrane. Following primary antibody incubation, detection was performed using either HRP-conjugated secondary antibodies (Pierce) or IR dye-conjugated secondary antibodies in conjunction with the Odyssey detection system (Li-cor).

## Abbreviations

MECs: mammary epithelial cells; IAP: inhibitor of apoptosis; XIAP: X-linked inhibitor of apoptosis; c-IAP: cellular- inhibitor of apoptosis; BIR: baculovirus IAP repeat; STS: staurosporine.

## Authors' contributions

TWO participated in the design of the study, carried out the cell culture experiments, collected the mammary gland samples and drafted the manuscript. FMF and JHF performed the immunoblotting and quantitative PCR analysis of the *in vivo *samples, as well as subsequent statistical analysis. FMF also contributed to drafting of the manuscript. JYC provided important discussion on the *in vivo *studies and did the experiment to compare IAP levels in isolated P18 and L7MECs. LB and JTH performed the quantitative PCR analysis of the adipocyte gene expression. CHS conceived the study, participated in its design and coordination, and helped to draft the manuscript. All authors read and approved the final manuscript.

## Supplementary Material

Additional file 1 **XIAP down-regulation in MECs is caspase-independent**. Primary MECs were cultured in complete media (lane 1), serum starved (lane 2), or serum starved in the presence of 100 μM zVAD, 10 ng/ml EGF or 5 μg/ml insulin (lanes 3, 4 and 5, respectively). **(A) **Whole cell lysates were immunoblotted with antibodies shown. **(B) **XIAP protein levels relative to calnexin were calculated using Li-cor Odyssey system. Click here for file

Additional file 2 **XIAP down-regulation in response to ECM-withdrawal**. **(A-B) **Primary MECs were left untreated (lane 1) or treated with 10 μM staurosporine (STS) (lanes 2-5). **(A) **Whole cell lysates were immunoblotted with antibodies shown (i). XIAP protein levels relative to ERK were calculated using Li-cor Odyssey system (ii). **(B) **Apoptosis was scored by counting the percentage of apoptotic nuclei. **(C) **Primary MECs were either left adherent (lane 1) or detached and re-plated onto poly-HEMA-coated dishes for 4 hr or 8 hr (lanes 2 & 3, respectively). Representative immunoblots (i) and relative XIAP protein levels (ii). Results shown represent the average of 3 experiments +/- S.E.M. (*** indicates p < 0.01; *indicates p < 0.05). Click here for file

Additional file 3 **XIAP down-regulation in the MEC line FSK7**. **(A-B) **FSK7 cells were cultured in completed media (lanes 1 and 4), serum starved (lanes 2 and 5) or serum starved in the presence of 10 μM Iressa (lanes 3 and 6). **(A) **Representative immunoblots (i) and relative XIAP protein levels (ii). **(B) **Apoptosis was scored by counting the percentage of apoptotic nuclei. **(C-D) **FSK7 cells were left untreated (lane 1) or treated with 10 μM staurosporine (STS) for times shown (lanes 2-5). **(C) **Representative immunoblots (i) and relative XIAP protein levels (ii). **(D) **Percentage apoptosis. **(E) **FSK7 cells were either left adherent (lane 1) or detached and re-plated onto poly-HEMA-coated dishes for 4 hr or 8 hr (lanes 2 & 3, respectively). Representative immunoblots (i) and relative XIAP protein levels (ii). Results shown represent the average of 3 experiments +/- S.E.M. (*** indicates p < 0.01; indicates p < 0.05). Click here for file

Additional file 4 **PCR primers used to detect IAP expression in mammary gland**. Click here for file

Additional file 5 **PCR primers used for quantitative PCR analysis of IAP transcript abundance **[45,46]. Click here for file
