# Supplementary figures and images for: Analysis of inhibitor of apoptosis protein family expression during mammary gland development
Source: BMC Dev Biol. 2010 Jun 28;10:71. doi: 10.1186/1471-213X-10-71 (PMC2905336; doi:10.1186/1471-213X-10-71)

# A)

Primary MEC

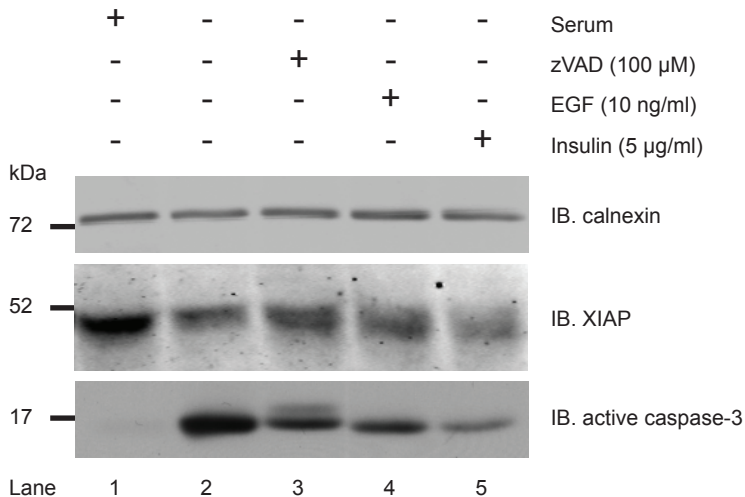

# B)

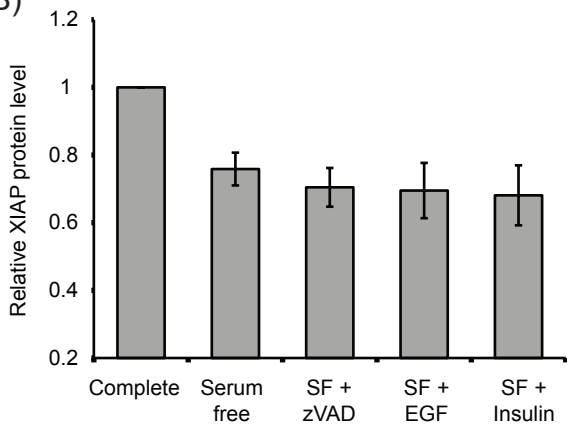

Supplement: Additional file 1 — XIAP down-regulation in MECs is caspase-independent. Primary MECs were cultured in complete media (lane 1), serum starved (lane 2), or serum starved in the presence of 100 μM zVAD, 10 ng/ml EGF or 5 μg/ml insulin (lanes 3, 4 and 5, respectively). (A) Whole cell lysates were immunoblotted with antibodies shown. (B) XIAP protein levels relative to calnexin were calculated using Li-cor Odyssey system. [file 1471-213X-10-71-S1.PDF]

A.

i)

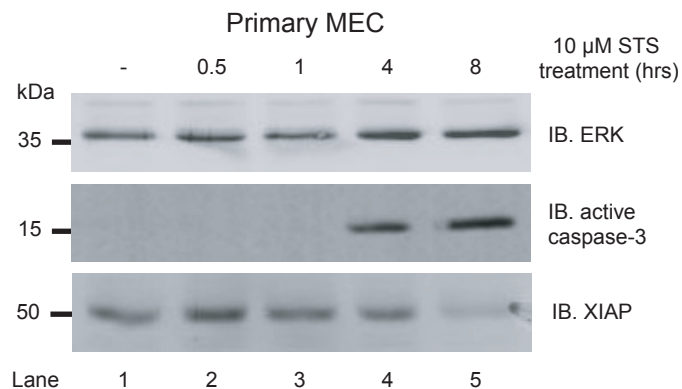

ii)

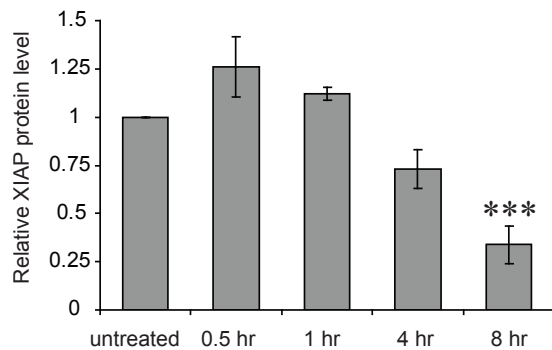

B.

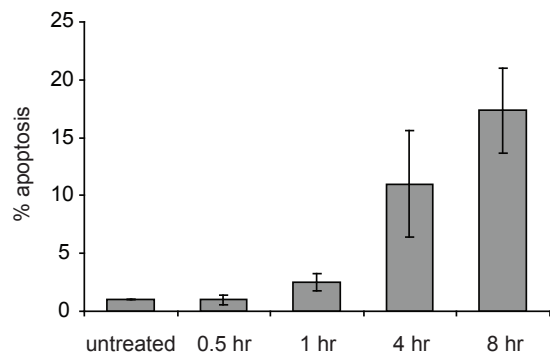

C.

i)

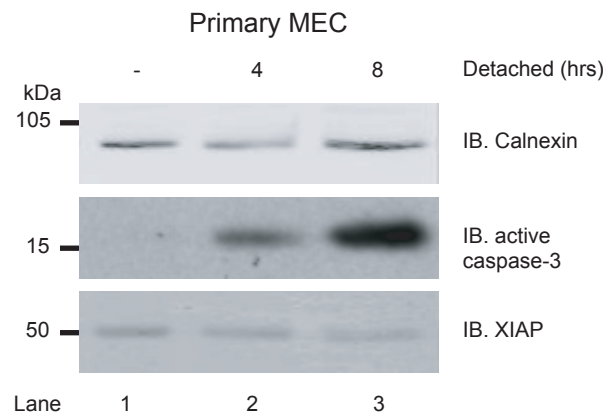

ii)

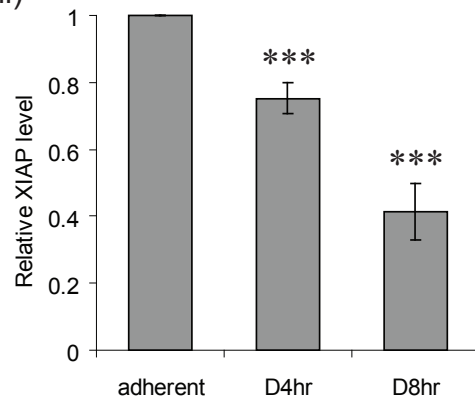

Supplement: Additional file 2 — XIAP down-regulation in response to ECM-withdrawal. (A-B) Primary MECs were left untreated (lane 1) or treated with 10 μM staurosporine (STS) (lanes 2-5). (A) Whole cell lysates were immunoblotted with antibodies shown (i). XIAP protein levels relative to ERK were calculated using Li-cor Odyssey system (ii). (B) Apoptosis was scored by counting the percentage of apoptotic nuclei. (C) Primary MECs were either left adherent (lane 1) or detached and re-plated onto poly-HEMA-coated dishes for 4 hr or 8 hr (lanes 2 & 3, respectively). Representative immunoblots (i) and relative XIAP protein levels (ii). Results shown represent the average of 3 experiments +/- S.E.M. (*** indicates p < 0.01; *indicates p < 0.05). [file 1471-213X-10-71-S2.PDF]

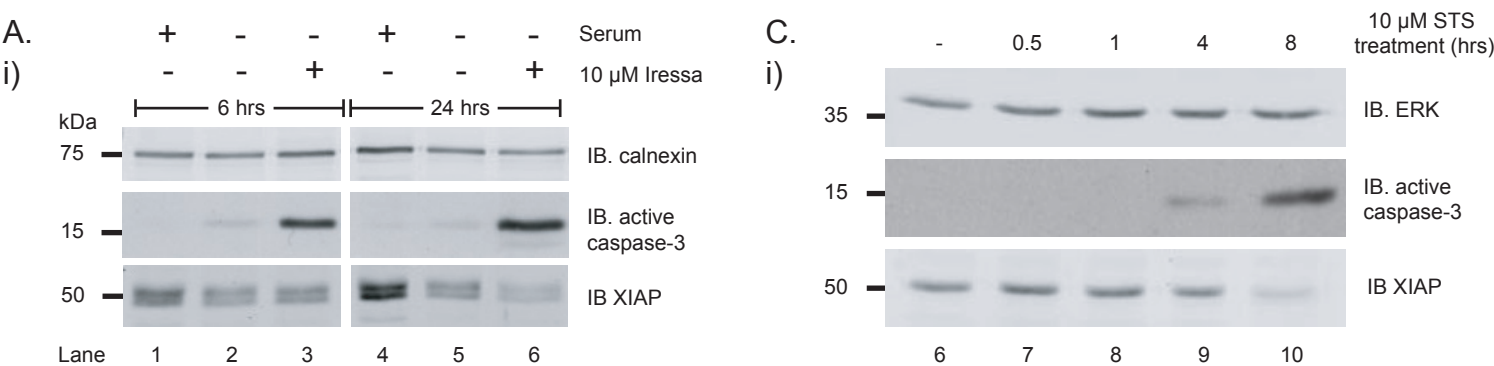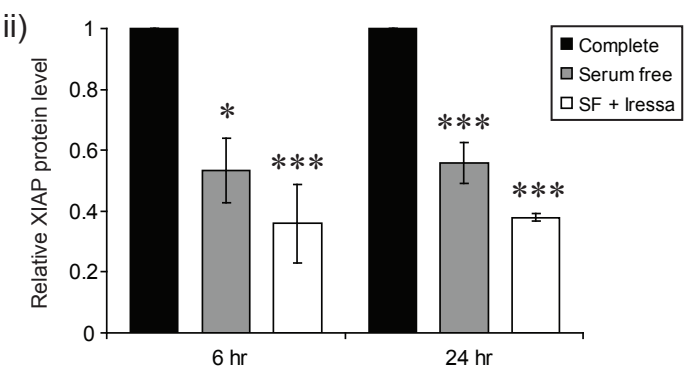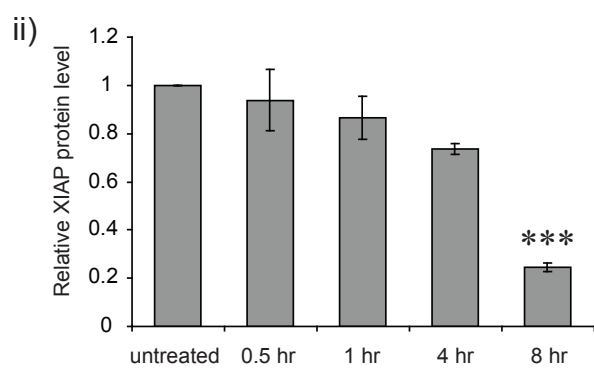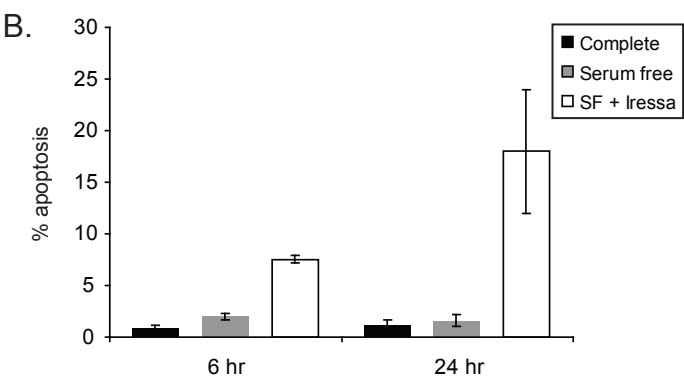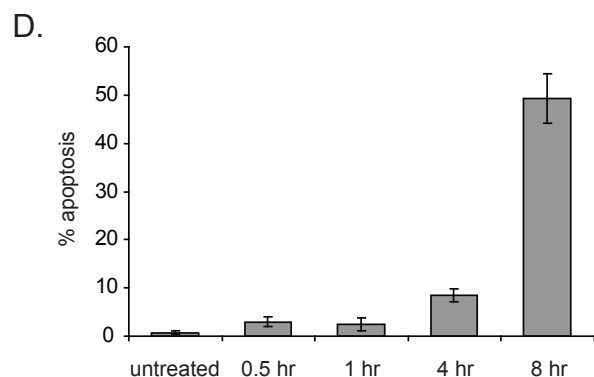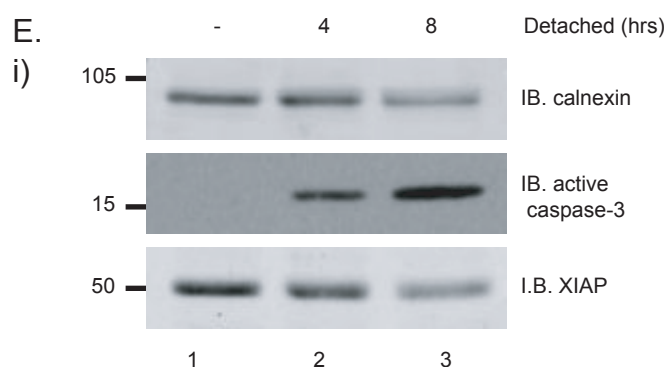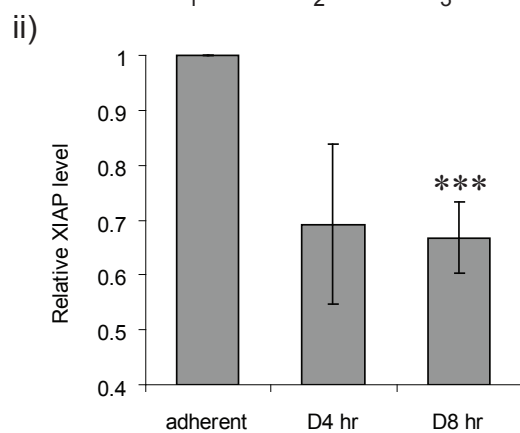

Supplement: Additional file 3 — XIAP down-regulation in the MEC line FSK7. (A-B) FSK7 cells were cultured in completed media (lanes 1 and 4), serum starved (lanes 2 and 5) or serum starved in the presence of 10 μM Iressa (lanes 3 and 6). (A) Representative immunoblots (i) and relative XIAP protein levels (ii). (B) Apoptosis was scored by counting the percentage of apoptotic nuclei. (C-D) FSK7 cells were left untreated (lane 1) or treated with 10 μM staurosporine (STS) for times shown (lanes 2-5). (C) Representative immunoblots (i) and relative XIAP protein levels (ii). (D) Percentage apoptosis. (E) FSK7 cells were either left adherent (lane 1) or detached and re-plated onto poly-HEMA-coated dishes for 4 hr or 8 hr (lanes 2 & 3, respectively). Representative immunoblots (i) and relative XIAP protein levels (ii). Results shown represent the average of 3 experiments +/- S.E.M. (*** indicates p < 0.01; indicates p < 0.05). [file 1471-213X-10-71-S3.PDF]
