# Supplementary material for: Analysis of inhibitor of apoptosis protein family expression during mammary gland development
Source: BMC Dev Biol. 2010 Jun 28;10:71. doi: 10.1186/1471-213X-10-71 (PMC2905336; doi:10.1186/1471-213X-10-71)
Supplement: Additional file 4 — PCR primers used to detect IAP expression in mammary gland. [file 1471-213X-10-71-S4.PDF]

Primer name	Sequence	Base length	T_m (°C)
BRUCE_for	5'-aat ggg atc ttg ttg cta gac act gc-3'	26	59
BRUCE_rev	5'-gat aag agc tgc tgt gcc tct gtg a-3'	25	60
c-IAP1_for	5'-caa aac tgt ctc cca gag act cgg-3'	24	64
c-IAP1_rev	5'-ctg aaa gca gag tct gta caa agc-3'	21	61
c-IAP2_for	5'-gtt gcg gaa act cca gga gga aag-3'	24	64
c-IAP2_rev	5'-cat gga cca tta gtc ttg ttc agg-3'	24	61
XIAP_for	5'-gtg agt gct cag aaa gat aat acg-3'	24	59
XIAP_rev	5'-taa cat gtg gtg ccc cac taa gac-3'	24	63
Surv_for	5'-atc gcc acc ttc aag aac tg-3'	20	58
Surv_rev	5'-tga ctg acg ggt agt ctt tgc-3'	21	58
NAIP_for	5'-ata agc caa caa ttc cca gat aag-3'	24	58
NAIP_rev	5'-gga aat ctg gta ttc tga act atc-3'	24	58
Omi_for	5'-cct gat ctc ctc ctt gct ttc c-3'	22	58
Omi_rev	5'-tgc agc ggt tag agg gac tt-3'	20	57
Smac_for	5'-cat tga cag tca gac cag agc-3'	21	57
Smac_rev	5'-cta aga acc agg cac aga gg-3'	20	56
GAPDH_for	5'-tga cat caa gaa ggt ggt gaa gc-3'	23	60
GAPDH_rev	5'-aag gtg gaa gag tgg gag ttg ctg-3'	24	60
