# Supplementary material for: Analysis of inhibitor of apoptosis protein family expression during mammary gland development
Source: BMC Dev Biol. 2010 Jun 28;10:71. doi: 10.1186/1471-213X-10-71 (PMC2905336; doi:10.1186/1471-213X-10-71)
Supplement: Additional file 5 — PCR primers used for quantitative PCR analysis of IAP transcript abundance [45,46]. [file 1471-213X-10-71-S5.PDF]

**Primer name**	**Sequence**	**Reference**
XIAP forward	GCACGGATCGTTACTTTTGGAACA	n/a
XIAP reverse	GTGGAAGCACTTCACTTTATCGCC	n/a
c-IAP1 forward	TGCCTGTGGTGGGAAACTGA	n/a
c-IAP1 reverse	GCTCGGGTGAACAGGAACA	n/a
c-IAP2 forward	TATTTGTGCAACAGGACATTAGGAGT	n/a
c-IAP2 reverse	CACATTCTTTCCTCCTGGAGTTTC	n/a
Adiponectin forward	AAGGACAAGGCCGTTCTCT	[45]
Adiponectin reverse	TATGGGTAGTTGCAGTCAGTTGG	[45]
Perilipin A forward	GAAGCATCGAGAAGGTGGTAGA	[46]
Perilipin A reverse	GCATGGTGTGTCGAGAAAGAG	[46]
β-actin forward	AAGTCCCTCACCCTCCCAAAAG	n/a
β-actin reverse	AAGCAATGCTGTCACCTTCCC	n/a
